# Diagnosis and Current Treatment of Aneurysmal Bone Cysts

**DOI:** 10.7759/cureus.53587

**Published:** 2024-02-04

**Authors:** Khalid A Bakarman

**Affiliations:** 1 Orthopaedic Surgery, King Saud University, Riyadh, SAU

**Keywords:** aneurysmal bone cysts, benign bone tumor, abc, curettage, bone tumor

## Abstract

The purpose of this review is to increase awareness about the evolution and development of current trends in the diagnosis and treatment of aneurysmal bone cysts (ABCs). ABCs are benign, but locally aggressive bone tumors that mainly affect children. ABCs comprise 1% of all primary bone tumors and occur most frequently during the first two decades of life. The diagnosis is made using a variety of imaging modalities and has the characteristic features of an expansile, radiolucent lesion that is often seen in the metaphyseal region of the bone and has fluid-fluid levels that are apparent on MRI. In the pediatric population, telangiectatic osteosarcoma and unicameral bone cyst (UBC) are the main differential diagnoses of an ABC. Giant cell tumors (GCTs) also include in differential diagnosis, which often manifest in patients older than 15 and do not penetrate the open physis although they develop after the physeal closure. Imaging alone cannot rule out telangiectatic osteosarcoma; therefore, a biopsy is recommended. A variety of treatment options have been described; traditionally, most patients are treated with curettage and bone grafting. Curettage alone, however, usually results in tumor recurrence following excision. A variety of adjuvants have been utilized with varying degrees of effectiveness to reduce the risk of local recurrence. When a cyst is in the pelvis, its location and size are such that surgery is a very risky option. Selective arterial embolization has significantly contributed to the development of effective treatments for these situations. Embolization or radiation, as well as denosumab therapy, are widely used as therapies for ABCs in anatomic locations where surgery would significantly increase morbidity.

## Introduction and background

The first formal description of an aneurysmal or bone cyst was given in 1942 by Jaffe et al. Both authors later added to their descriptions of this lesion, which led to the name "Jaffe-Lichtenstein disease" for the condition [1]. The word bone cyst was used to indicate how the disease often appears as a cavity filled with fluid. The term "aneurysmal" was chosen to represent the blown-out, distended contour of the affected bone [2]. Only in a descriptive sense should the term "aneurysmal bone cyst" (ABC) be used. Due to the lack of understanding of pathophysiology and causative pathways, attempts to identify a genetic or neoplastic origin have never been successful [3-6]. Recent studies on recurrent chromosomal translocations associated with the USP6 gene have shown that ABCs is a clonal neoplastic process; about 65-70% and 30% of individuals with ABC, had USP6 rearrangements and CDH 11-USP6 fusions, respectively [4,7].

In the most recent WHO classification of Tumors of Bones, 2020, the terms "ABC" and "ABC-like changes," which are present within some pre-existing primary bone neoplasms, are suggested in place of "primary ABC" and "secondary ABC," respectively [8]. About 70% of cases of "ABC" have no underlying bone abnormalities regarded as primary bone lesions, whereas the remaining 30% of "ABC-like changes" have primary bone abnormalities. In his original work and a subsequent article, Jaffe proposed that the secondary phenomenon arises from a hemorrhagic "blow-out" in a preexisting lesion [1]. Liechtenstein et al. [1] proposed that the lesion was caused by a "local circulatory disturbance," noting that, while the precise cause of this vascular disturbance is unknown, it could be thrombosis of a large vein or an abnormal arterial-venous connection [8,9]. Neither the histogenesis nor the precise nature of aneurysmal bone cyst (ABC) is well known at this moment. It is a highly vascular lesion that develops in response to impaired local hemodynamics or "arteriovenous fistula." There are fibrous elements, macrophages, large cells, and bone islands among the lining cells of the cyst [10-14]. The histological hallmark of these lesions is the presence of blood-filled cystic spaces lacking an epithelial or endothelial lining. Three primary histology elements are recognized: (1) a cellular component consisting of large multinucleated cells with characteristics similar to those of osteoclasts, (2) a fibrillar component made up of collagenous extracellular matrix, and (3) a component known as an osteoid made up of organic bone matrix deposited by osteoblasts [15]. Numerous studies have found that those with preexisting lesions are more likely to develop ABCs due to hemorrhagic degradative processes. These other lesions include osteoblastoma, angioma, chondroblastoma, telangiectatic osteosarcoma, non-ossifying fibroma, fibrous dysplasia, chondromyxoid fibroma, and giant cell tumors. Many researchers have concluded that ABCs develop as a result of underlying vascular disorders [16-19]. Gutierrez et al. [20] found that ABC life changes were more often associated with giant cell tumors and chondroblastoma, both of which typically affect the epiphysis.

According to the findings of Rosenberg et al. [21], Panoitsakopoulos et al. [22], Oliveria et al. [23-25], and Dal Cin et al. [26], chromosomal translocation t (16;17) (q 22; p13) is a recurrent alteration in ABCs, suggesting that at least some of these lesions are neoplastic. Dal Cin et al. [26] studied two people with ABCs, one with solid ABC and one with extraosseous ABC. They discovered nonrandom rearrangements of the chromosome bands 16q22 and 17p13. This suggests that all ABCs share a common origin. Growth hormone affects skeletal growth and is mediated by the insulin-like growth factor IGF-I pathway, which is overexpressed in several tumors, including osteosarcoma [27]. In the study by Leithner et al., they observed overexpression of the insulin-like growth factor IGF-I in 19 of 19 individuals with ABCs, suggesting that IGF-I may potentially play a role in the pathogenesis of this tumor [27]. Solid ABC is an uncommon form of an ABC. Clinically and radiologically, it looks like a conventional form of an ABC lesion. In 1983, Sanerkin et al. [28] were the first to report this variant of ABC, which is best described as a "solid type of aneurysmal bone cyst" that contains a reticulated lacy, chondroid-like substance that differs from a conventional vascular or cystic cavity.

In the literature, this condition affecting the mandible and maxilla, as well as the short tubular bones in the hands and feet, is referred to as a "giant cell reparative granuloma," a term that was first used to describe a nonneoplastic hemorrhagic lesion by Jaffe [29] in 1953 and Lorenzo et al. [30] in 1982. Ackerman et al. in 1962 [31] described similar lesions in the phalanges as "giant cell reactions." The terms "solid variant" ABC and "giant cell reparative granulomas" have been used interchangeably in the literature because they exhibit the same histologic characteristics. The term "solid cyst" has drawn criticism from certain authors for being ambiguous [32-34]. The absence of blood-filled cystic cavities is the primary histological difference between the solid variant and ABC. The mandible and the posterior part of the vertebral bodies have both demonstrated solid ABC; on the other hand, cranial bones, facial bones, and the nasal cavity have all been documented to develop giant cell reparative granulomas [35].

**Figure 1:**
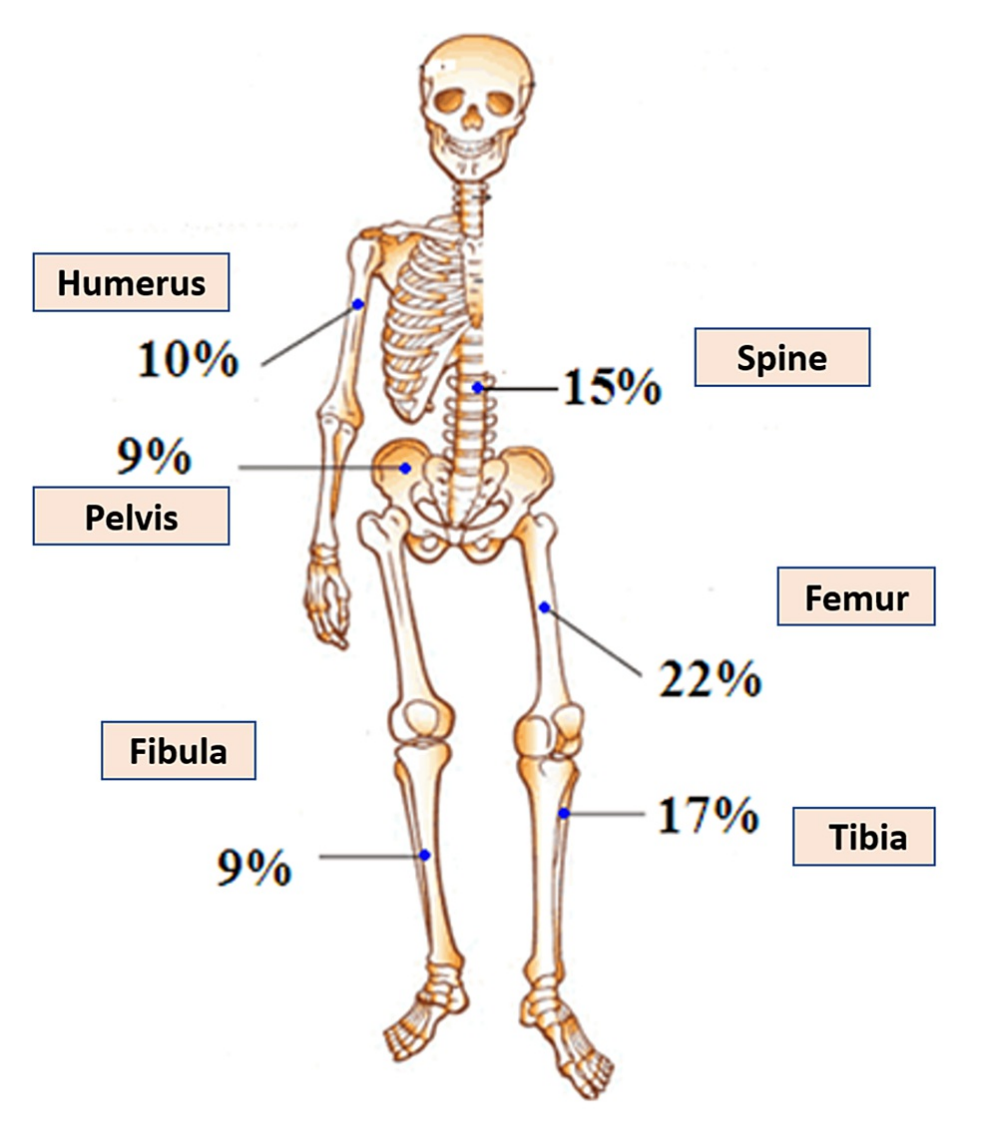
Distribution of sites of lesions in patients with aneurysmal bone cysts Source: Khalid A. Bakarman (author)

ABCs account for around 1% of all bone tumors [36]. Primary ABC is a rare disorder that affects 1.4 out of every million individuals [37]. Even though ABC may develop at any age, it most frequently occurs during the first and second decades of life. Several demographic studies [13,38] and data from Cottalorada et al. [39] on 411 children with primary ABCs revealed that the femur (22.3%), tibia (17.4%), and spine (15%) were the most commonly reported ABC locations. Although ABCs may affect any skeletal site, they are most often seen in the metaphyseal region of long bones (Figure 1).

## Review

Investigation and diagnosis

After a comprehensive history is obtained, a full physical examination is carried out. To determine the skeletal computed tomography (CT) scans are taken. Magnetic resonance imaging (MRI) is used to evaluate the extent of the lesion to the soft tissues and the intramedullary damage. The radiologist establishes a diagnosis or a differential diagnosis based on the location, age, and radiologic characteristics of the lesion. However, an accurate histological evaluation with imaging findings correlation is required for a definitive diagnosis. These often lend support to the diagnosis of ABCs.

**Figure 2:**
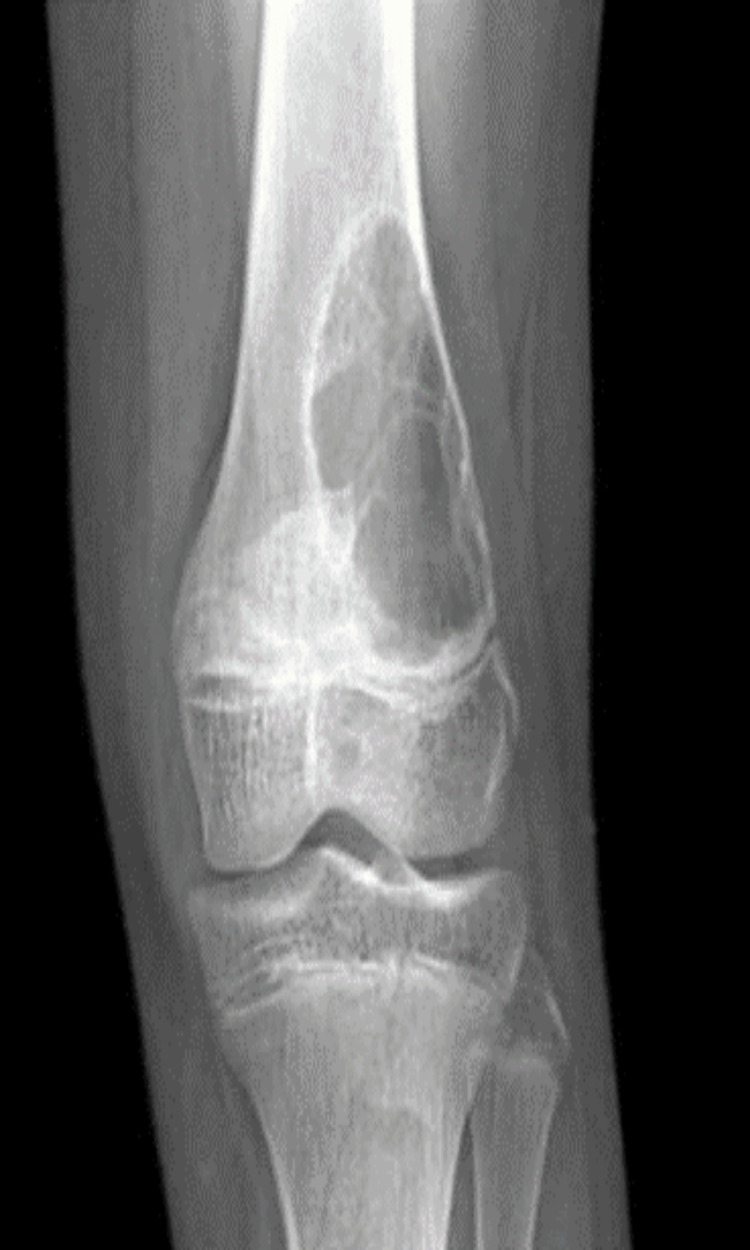
The distal metaphysis of the left femur has an eccentric, well-defined lytic lesion with a sclerotic margin and a narrow zone of transition with no bony expansion. There is no soft tissue component, periosteal reaction, or cortical breakthrough The image belongs to the author's patient.

An eccentric lytic expansile lesion that frequently has trabeculation within the lesion surrounded by a thin layer of cortical bone and subperiosteal new bone formation is what Jaffe [2] defined as the characteristic radiographic feature of ABCs. Depicted in Figure 2 is the author's own patient. In about 80% of the cases, the cyst in long tubular bones is eccentric in the metaphyseal region, but in short, tubular bones, the cyst may occur more centrally. In the spine, the pedicles, transverse process, and spinous process are the most posterior elements of the spine that are most frequently affected. The radiographic characteristics of ABC are atypical and exhibit close similarities to those of other benign cystic lesions. Additionally, ABC may be secondary to various benign and malignant disorders.

In their multiple publications [40-42], Capanna et al. classified tumor morphology into five categories that can be used to characterize the radiographic and gross appearance of ABCs. Type I lesions are well-contained, centrally located, and either lack an outline or have a very mild expansion. Type II lesions exhibit considerable expansion and cortical thinning and involve whole bone segments. Type III is an eccentric, metaphyseal lesion that often only affects one cortex. Type IV lesions are the least frequent subperiosteal lesions, which grow away from the bone. Type V lesions are periosteal lesions that grow peripherally and eventually penetrate the underlying cortex.

**Figure 3:**
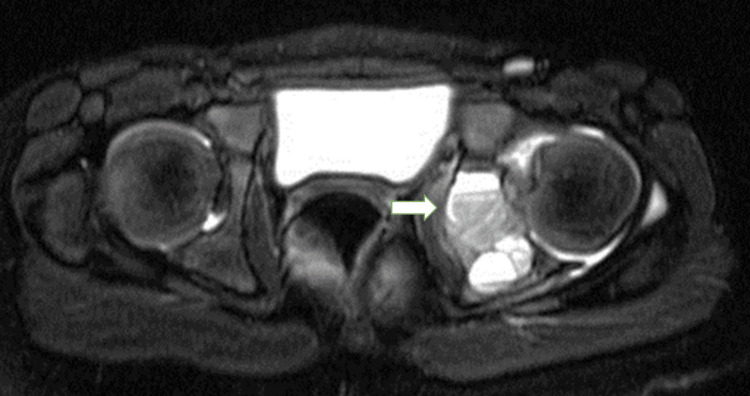
MRI axial images of an aneurysmal bone cyst of a six-year-old girl's left hip, showing septation at the posterior acetabulum with a fluid-fluid level The image belongs to the author's patient.

CT scans are used to more precisely identify lesions once they have been found on radiography. This is particularly beneficial for lesions located in regions such as the pelvis or spine, where the bony structure is complex and cannot be thoroughly investigated with simple films. A CT scan typically indicates an interrupted cortex, and the surrounding soft tissue mass is usually smooth, sharp, and well-defined, suggesting an intact periosteum. Hudson [43] reported the findings from 17 ABC CT scans, noting that fluid-fluid levels were seen in six of the cases (35%). He emphasized the need to employ a narrow window setting to study such scans to delineate lesions and narrow the differential diagnosis of ABCs by demonstrating multiple fluid levels within the cystic areas. Although the findings of MRI and CT scans are comparable, MRIs are more accurate in depicting fluid levels (Figure 3).

This lesion is characterized by a multicystic appearance, a hypointense rim, contrast-enhancing cyst walls, double-density fluid levels, and adjacent soft tissue edema [44,45]. Additionally, MRIs may more accurately depict the extent of the lesions and be useful in differentiating between an ABC and an atypical osteosarcoma or a telangiectatic osteosarcoma, both of which can resemble ABCs radiographically [46].

Biopsy

When an ABC is typical and all staging studies support that diagnosis, some surgeons opt for a "one-stage" approach without performing a biopsy prior. A biopsy may be performed as part of the definitive surgical operation. If there is doubt in the diagnosis, a tissue diagnosis is necessary. A biopsy of a suspected ABC should be discussed with the treating physician to establish the best strategy that does not risk limb salvage [36-47]. An ABC requires a precise and timely diagnosis because both benign and malignant tumors are on the differential diagnosis list. Osteolytic expansile lesions on plain X-rays and hemorrhagic fluid-fluid levels within cystic cavities on MRI are diagnostic features of telangiectatic osteosarcoma, which must be distinguished from ABCs.

**Figure 4:**
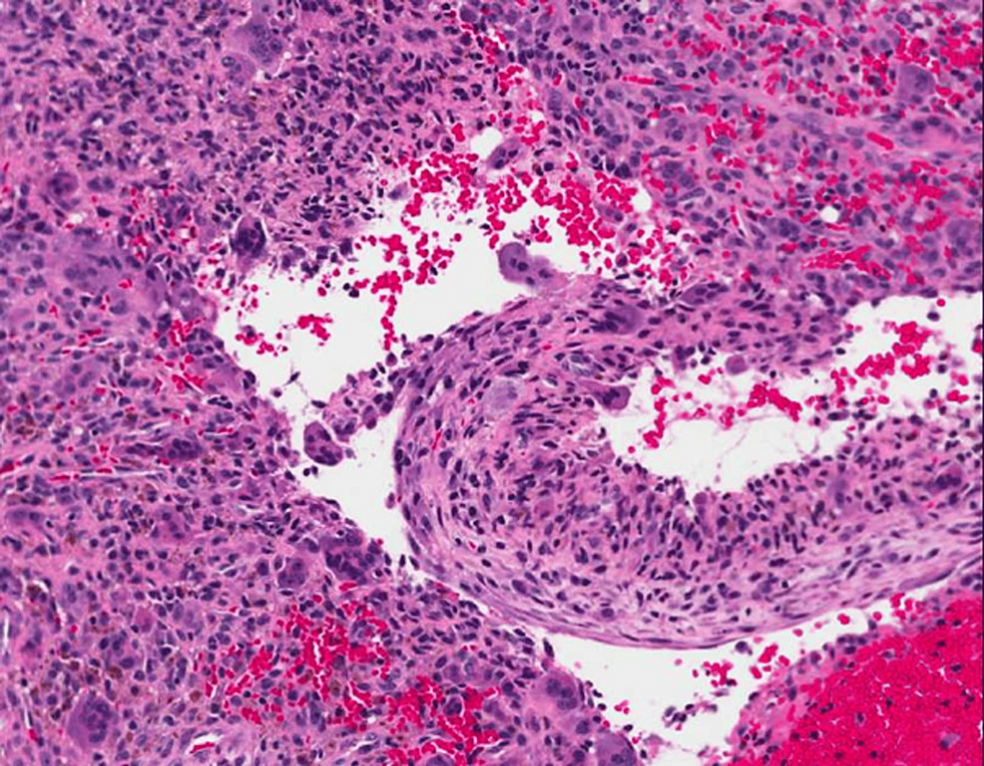
High-power histology of an excised aneurysmal bone cyst shows the characteristic irregular vascular spaces and septa containing giant cells The image belongs to the author's patient.

Tomino et al. [48] reported 51 cases of ABC that were first identified based on a radiological examination. However, following the biopsy, 17 of the cases were found to be those of a unicameral bone cyst. The presence of bloody liquid alone is not sufficient to make the diagnosis of an ABC; nevertheless, this can also be seen in fractured solitary bone cysts. On the other hand, clear liquid is frequently seen in solitary bone cysts. However, it is possible to find clear liquid in an inactive ABC. UBC and ABC are frequent benign lesions with similar clinical and radiological characteristics that affect comparable populations. In UBC, fractures are the most common complication. In ABCs, they focus more on the lesion's propensity for osteolysis and how it affects growth. In ABCs, a biopsy is necessary to rule out telangiectatic sarcoma [49]. Needle biopsies might be problematic at times because the material that is collected may be composed primarily of blood components. In many cases, the diagnosis can only be determined after an open biopsy and a frozen section have been performed. An ABC is usually composed of cavernous or slit-like hemorrhagic areas and is divided by a fibrous septum comprising fibroblasts, histiocyte-like cells, and multinucleated giant cells. Histologically, ABCs have a characteristic appearance [50]. The author derived the histological image in this study from a patient under his care, providing an insightful perspective into the specific pathological features observed in their clinical practice (Figure 4).

Various forms of therapy

Several different therapeutic strategies have been explored. However, the appropriate treatment is still a matter of debate. En block excision has the lowest recurrence rates with the highest morbidity. Whether or not a local adjuvant is used, intralesional surgical procedures are regarded as the standard of care [15]. Radiation therapy is currently reserved for tumors deemed irresectable because of the risk of sarcoma induction [51], when the tumor is located in an expandable bone (such as the fibula or rib) resection is recommended, with or without reconstruction [52]. Several authors have described adjuvant therapy in an attempt to reduce an unacceptably high recurrence rate. Adjuvants such as cryotherapy [53], polymethylmethacrylate [54], calcitonin and steroid injections [16,52,54], alcohol injections [54], and arterial embolization [55] are commonly employed. Percutaneous sclerotherapy has become a useful option for treating ABCs in recent years [52,53]. There have also been reports of ABCs healing spontaneously. It is difficult to determine the true incidence of spontaneous healing because the majority of ABCs are treated after diagnosis. After the biopsy, spontaneous healing has also been observed; this could be because the balance within the blood-filled cystic areas has altered in certain circumstances, particularly in individuals with small lesions and in locations with a very low risk of fracture. A conservative approach to ABC with close radiological follow-up can be considered [47,56].

Radiotherapy

In radiation therapy, external beam radiation is used causing damage to the nuclear DNA to induce cell death. Radiotherapy is often used for treating malignant tumors, although it has also been utilized in the past for ABCs, as adjuvant therapy in cases of recurrence and for incurable lesions. Radiation therapy carries several risks, including radiation-induced sarcoma, physeal arrest, and injury to the gonadal tissue [11,12]. In the case of ABC of the spine, Capanna et al. [41] compared radiotherapy to surgery and observed that, when radiotherapy was used as the only method of treatment, three of six patients developed a local recurrence, with two of those cases, resulting in patient death. On the other side, Marcove et al. [53] found a controlled rate of more than 90% when radiotherapy was administered, but they also reported that one out of 11 patients developed a radiation-induced sarcoma. Feigenberg et al. [56], in their series of patients with ABC of the spine treated with radiation, identified various adverse effects, including dorsal kyphosis due to vertebral body collapse, but no secondary malignancy was found. Similarly, Boriani et al. [57] reported that a substantial incidence of late axial deformity was associated with the combination of curettage and radiation in a spinal ABC. The effectiveness of radiotherapy in treating ABC is questionable, particularly because of the possibility of significant adverse effects. However, intralesional injection of radioisotopes that create ionizing radiation and ablate tissues has emerged as a safe and effective option. Bush et al. [58] reported that five axial skeleton ABCs were successfully controlled with no detrimental consequences.

Selective Arterial Embolization (SAE)

SAE may be used to reduce interoperative blood loss. It has also been shown to be an effective therapeutic strategy to cut off the nutrient supply and change the hemodynamics of the lesion without affecting the vascularity of adjacent tissues or structures. If the size or location (the spine or pelvis) of the lesion makes treatment challenging, this method of treatment is effective. Skin necrosis and transient paresis are common complications that affect 5% of patients [59]. Donati et al. [60] noted a high rate of local control in ABCs of the sacrum that was treated solely with embolization. Regardless of these results, SAE is still a limited option since these lesions could lack well-defined feeding arteries and there is a risk that the arterial supply to the surrounding normal tissue might be embolized [58,60]. The procedure is technically challenging, and complications are not insignificant. Another study by Andreani et al. [61] did not advise for SAE, particularly ABCs of the thoracic spine supplied by the artery of Adamkiewicz, whose embolization may result in permanent neurologic damage due to cord ischemia.

Sclerotherapy

Percutaneous sclerotherapy has emerged as an excellent approach to treating ABCs in recent years. Sclerosants cause damage to the vascular endothelium, promote thrombosis in small blood vessels, and trigger a series of intricate processes that culminate in the repair of the lesion. An alcoholic zein solution is a radiopaque solution, and when used during sclerotherapy procedures, it may be detrimental to the adjacent tissues [62]. Polidocanol is a safe and effective sclerotherapy for ABCs, with few side effects. Rastogi et al. [63]. reported a clinical response of 84.5% of 72 patients, with an average follow-up of 34 months and three injections given to each patient on average. Vershney et al. [64] compared the effectiveness of a curettage, high-speed burr, and bone graft in a randomized trial with polidocanol sclerotherapy. The recovery rate for curettage was 84.8%, whereas that for polidocanol was 93.3%; the difference was not statistically significant.

Several other agents that were injected into ABCs were found to have an effect; doxycycline has been used successfully for the percutaneous treatment of ABCs and most likely has a similar mode of action as sclerosants [65]. Injections of alcohol have a negligible risk of adverse effects, However, only 59% of the lesions showed a satisfactory response [66]. Additionally, bone marrow injection has frequently been shown to speed up healing [67].

In summary, the percutaneous injection of polidocanol is a straightforward, risk-free therapy with a high cure rate. The procedure does require technical expertise with local/general anesthesia and is done under fluoroscopic guidance with added radiation exposure. It is not completely risk-free as inflammatory reaction/anaphylaxis can still occur. The cure rate is good and comparable to other options. A sentence can be stated as "percutaneous injection of polidocanol is a simple procedure, safer with an excellent cure rate."

Intralesional Surgical Procedures With or Without Local Adjuvants

Currently, curettage and bone grafting, with or without adjuvant therapy, are the accepted approaches for treating ABCs with variable recurrence rates of up to 59% in some series [16]. Curettage is the procedure in which there is a formation of small fenestration or big cortical windows in the cyst to complete saucerization. Saucerization is a technique in which a new subperiosteal bone is removed along with connected cysts [16,47].

Phenol

Phenol, commonly referred to as carbolic acid, is a precursor to many other chemicals, including analgesics and polymers. After curettage, the remaining neoplastic cells are destroyed with this therapy [68,69]. Recurrence rates following curettage with phenol were 7% compared to 41% after curettage alone, according to a retrospective case series published by Capanna et al. [69]. Keçeci et al. [70] in a comparative analysis of 85 patients found no statistically significant differences between curettage alone, curettage combined with a high-speed burr, or curettage combined with a high-speed burr and phenol or alcohol.

Bone Cement (Polymethylmethacrylate, PMMA)

Bone graft reconstruction is widely utilized after curettage to enhance osseous healing of the resultant cavity. PMMA cement may be used to stabilize pediatric benign bone lesions immediately after surgery. Due to its heating effect, PMMA cement serves as an adjuvant that effectively prevents recurrence [71]. In regard to the effectiveness of cement in reducing recurrence, the evidence is contradictory. Ozaki et al. [72] demonstrated that curettage and cementing reduced recurrence as compared to curettage and grafting alone by 17% and 37%, respectively. Mankin et al. [50] and Wallace et al. [73] compared the results of using cement or bone grafting to treat benign bone lesions in children and found that the rates of complications and recurrence were the same whether cement or bone graft was used.

Cryosurgery

Cryosurgery involves pouring liquid or aerosolized nitrogen into the cavity following curettage to create cytotoxic freezing temperatures to affect the ABC lesion [71]. Even though cryosurgery has a low recurrence rate, it is not widely adopted. This is most likely because of its unfamiliarity and risk for complications, which include post-operative fracture and skin necrosis/wound infection rates of up to 14% and 8%, respectively [55,74]. Marcove et al. [53] observed a 17.6% recurrence rate after curettage and that liquid nitrogen pouring was reduced to 4% after a second cryosurgery. Schreuder et al. [74] treated a group of 80 ABC patients with curettage and nitrogen spray, and they noted a 3.7% recurrence rate. The advantage of cryosurgery over local excision is that the supporting function of the bone is preserved, limiting the need for reconstructive surgery.

Argon Beam Coagulation

The argon beam of laser therapy is a more sophisticated technique that induces desiccation and coagulation by the unit polar electrical current into the tissue. Directing argon beam therapy at an ABC lesion following curettage has been shown to reduce recurrence rates [75]. According to Cummings et al. [76], curettage followed by the application of the argon beam to the edges of the residual lesion resulted in a recurrence rate of 0%.

Steffner et al. [77] found that the postoperative fracture rate with the argon beam was 12.5%, resulting in desiccation and osteonecrosis from the argon beam, as compared to none and the curettage group. They also compared the recurrence rate of curettage, high-speed burr, and the use of argon beam coagulation, which resulted in 7.5%-20.6%, respectively. Surgeons are not familiar with the technique, and the operating room is not equipped with argon beam technology, which would explain why argon beam coagulation has not gained popularity.

High-Speed Burr

After intralesional resection of an ABC lesion, a high-speed burr can be used to augment curettage by mechanical disruption of the lesion to the level of the circumscribing bone [71]. When a high-speed burr was used, Wang et al. [78,79] achieved a greater control rate of approximately 97%. Gibbs et al. [55] performed curettage with a high-speed burr without the use of any adjuvant on the 34 patients and achieved approximately a 90% control rate after a median follow-up of 7.2 years.

Summary: When combined with curettage, adjuvants ought to increase local control rates. However, it must be remembered that not all of the agents mentioned above are readily available. Cryosurgery and argon beam coagulation are only available at a small number of centers. They may also lengthen the surgical procedure, and it is important to remember that comparable control rates have been achieved with less invasive techniques as well.

En Block Excision

En block excision, which is also called “complete resection,” has the lowest risk of recurrence. Complete resection may create a defect in the bone that needs to be addressed, which could be challenging depending on the location of the lesion [50,51,71]. En block excision is the best treatment option for lesions that develop in expandable bones (clavicle, rib, proximal fibula, pubic ramus) or for recurrent lesions and lesions that do not respond to less invasive treatments [51,80].

In children, subperiosteal resections can be performed, and the periosteum that is left over will help the bone become stronger over time. However, in adults, it is technically not feasible to perform because the periosteum cannot be separated [81]. After a wide resection, there was no local relapse in the series by Campanacci et al. [51], Vergel de Dios et al. [82], and Lampasi et al. [83] reported that ABCs of the distal fibula did not recur after complete resection, but they pointed out that this method has a higher risk of complications than intralesional excision, particularly in the context of similar functional levels. Abuhassan et al. [84] and Mostafa [85] noticed that complete subperiosteal resection has a high local control rate (100%) in cases of ABCs of the distal fibula. It also retains the periosteum, allowing the patient to recover quickly.

Medical Treatment

In the literature, pyrophosphate analogs, also known as bisphosphonates, have been used as a conservative treatment for ABC [15,69-81]. Bisphosphonates inhibit osteoclast-mediated bone resorption because of a similar molecular structure to pyrophosphate. Additionally, the antineoplastic properties of bisphosphonates inhibit angiogenesis by inducing tumor cells to undergo apoptosis and limiting tumor cell adherence and invasion [81]. A study by Cornelis et al. [86] observed pain relief with bisphosphonate treatment for benign bone tumors that are symptomatic and inoperable, including ABCs.

RANKL, which stands for receptor activator of nuclear factor kappa B ligand, is a signaling pathway that is crucial for regulating and remodeling bone by osteoclast activation [17,87].

RANKL is expressed by a wide variety of benign and malignant bone neoplasms, and there is significant evidence that RANKL expression is higher in ABCs. RANKL is selectively inhibited by the human monoclonal antibody denosumab [87]. Denosumab may be an effective neoadjuvant treatment for ABCs and other osteolytic bone diseases, according to Dubory et al. [88]. It has been shown that denosumab can reduce tumor size, which can minimize the potential morbidity of surgical procedures. Denosumab can be utilized when surgical interventions and/or embolization are ineffective or impractical [80].

Table 1 summarizes the various methods that have been developed along with their advantages and disadvantages. Curettage is preferred by many surgeons; nonetheless, there is a significant risk of complications and associated morbidity. Curettage should be reserved for lesions that do not respond to sclerotherapy and preferably with a high-speed burr to reduce the chance of local recurrence. As more institutions gain access to our argon lasers, this technique appears to be gaining popularity because of its ease of use and minimal adverse effects. In anatomic locations where surgery would substantially increase morbidity, embolization or radiotherapy are the most often used therapies. Radiation therapy should not be used as the first line of treatment for the vast majority of ABCs due to its high failure rate and increased risk of malignancy. Polidocanol sclerotherapy, on the other hand, is an appropriate first-line therapy.

**Table 1:** Prevalent therapies for aneurysmal or bone cysts, with indications, advantages, and disadvantages

Treatment	Major indications	Advantages	Disadvantages
Radiotherapy	Lesions that are not amenable to other methods	Non-invasive	Significant morbidity, growth disturbances, and potential for secondary malignancies
Embolization	When difficult-to-access lesions are treated as an adjunct to surgery or sclerotherapy	Good local control	Risk of neurological consequences, the requirement for equipment, and expert operator
Sclerotherapy	The primary treatment for all ABCs	Can be done on an outpatient basis, good functional outcomes, cost-effective	Requires several procedures
Intralesional excision	Surgically accessible ABCs, especially after unsuccessful sclerotherapy	In general, a single procedure is curative, and an outpatient procedure is not practical	Morbidity and adverse effects associated with the procedure (bleeding, growth disturbances, infection risk)
En bloc excision	ABCs in expendable bones	Excellent local control rate	Morbidity and adverse effects associated with the procedure (bleeding, growth disturbances, infection risk)

## Conclusions

ABCs are aggressive benign tumors with a high rate of recurrence, making them challenging to treat. A biopsy is recommended when an ABC is suspected to confirm the diagnosis. The optimal method to manage ABCs is still a matter of debate. These benign lesions are occasionally resolved spontaneously, and aggressive surgery should be avoided initially.
